# Cut instance mixing: A domain-specific data augmentation method applied to gastrointestinal lesion detection

**DOI:** 10.1038/s41598-026-42138-2

**Published:** 2026-03-04

**Authors:** Alexandre Neto, Eduarda Almeida, Diogo Libânio, Mário Dinis-Ribeiro, Miguel Coimbra, António Cunha

**Affiliations:** 1 Centro de Investigação em Engenharia Biomédica (C-BER), Instituto de Engenharia de Sistemas e Computadores, Tecnologia e Ciência (INESC TEC), Porto, 4200-465 Portugal; 2 Escola de Ciências e Tecnologia, Universidade de Trás-os-Montes e Alto Douro, Vila Real, 5001-801 Portugal; 3 CIDES/CINTESIS - Departamento de Ciências da Informação e da Decisão em Saúde/Centro de Investigação em Tecnologias e Serviços de Saúde, Faculdade de Medicina, Universidade do Porto, Porto, 4200-319 Portugal; 4 Faculdade de Ciências, Universidade do Porto, Porto, 4169-007 Portugal

**Keywords:** Cut-and-paste, Data augmentation, Deep learning, Gastrointestinal endoscopy, Multiple-image combination, Colonoscopy, Gastrointestinal system, Oesophagogastroscopy, Biomedical engineering, Gastroenterology, Health care, Engineering, Computational biology and bioinformatics, Computational models, Data processing, Machine learning

## Abstract

Early detection of gastrointestinal lesions such as intestinal metaplasia (IM), dysplasia, and polyps remains challenging due to their subtle appearance and the scarcity of well-annotated medical image datasets. To address this limitation, we introduce Cut Instance Mixing (CIM), a domain-specific data augmentation method designed to generate anatomically plausible lesion-containing images through the identification of biologically relevant regions of interest and seamless lesion blending using Poisson image editing and gradient-based mixing. CIM was evaluated across three distinct endoscopic datasets (IM, dysplasia, and polyps) using a ResNet50 classifier and five-fold cross-validation. The proposed method consistently outperformed state-of-the-art augmentation techniques. In IM classification, CIM with α = 0.8 achieved the highest performance (AUC: 0.879, Accuracy: 0.823), surpassing MixUp, CutMix and random copy-paste. In dysplasia detection, CIM reached near-perfect results (AUC: 0.997, Accuracy: 0.966), and demonstrated strong generalization on an external polyp dataset (AUC: 0.830, Accuracy: 0.769). Grad-CAM analyses further confirmed that CIM preserves clinically relevant features, improving model attention on lesion regions. These findings demonstrate that CIM enables the generation of realistic and biologically coherent synthetic samples, effectively mitigating data imbalance and enhancing classification robustness. The method is architecture-agnostic and broadly applicable to tasks requiring anatomically consistent augmentation, providing a promising direction for improving deep learning systems in gastrointestinal imaging.

## Introduction

Gastrointestinal (GI) lesions, including gastric cancer, pose significant clinical concerns, with gastric cancer being the fifth most common and fourth deadliest cancer globally. Early detection of GI lesions is critical for effective care. While endoscopy is the gold standard, identifying precursors like intestinal metaplasia and dysplasia can be challenging due to their subtle and variable features^1–4^.

Deep learning (DL) has revolutionized computer-aided diagnosis systems, offering significant benefits for GI endoscopy by enhancing lesion detection, localization, and characterization. These advancements are particularly valuable for identifying challenging conditions like intestinal metaplasia (IM) and dysplasia. However, the effectiveness of DL models in gastroenterology is limited by the scarcity of high-quality, annotated medical image datasets. Data collection faces challenges such as high costs, labour-intensive manual annotation, and limited dataset diversity, especially for underrepresented lesion types, making it difficult to develop robust and accurate DL algorithms^5,6^.

Data augmentation through synthetic data generation helps overcome the challenge of limited annotated datasets in gastroenterology. By creating diverse, high-quality synthetic images, this approach addresses the scarcity of data for underrepresented conditions like IM and dysplasia. It can improve DL model performance, reduce the need for costly manual annotations, and support more accurate detection of GI lesions^7–9^.

Our study focuses on developing a new data augmentation method capable of generating an unlimited number of pre-annotated samples. The approach aims to address data scarcity in gastroenterology by producing diverse and high-quality datasets for different DL tasks through the controlled combination of lesion characteristics and anatomically plausible placement.

### State-of-the-art data augmentation techniques

**Fig. 1:**
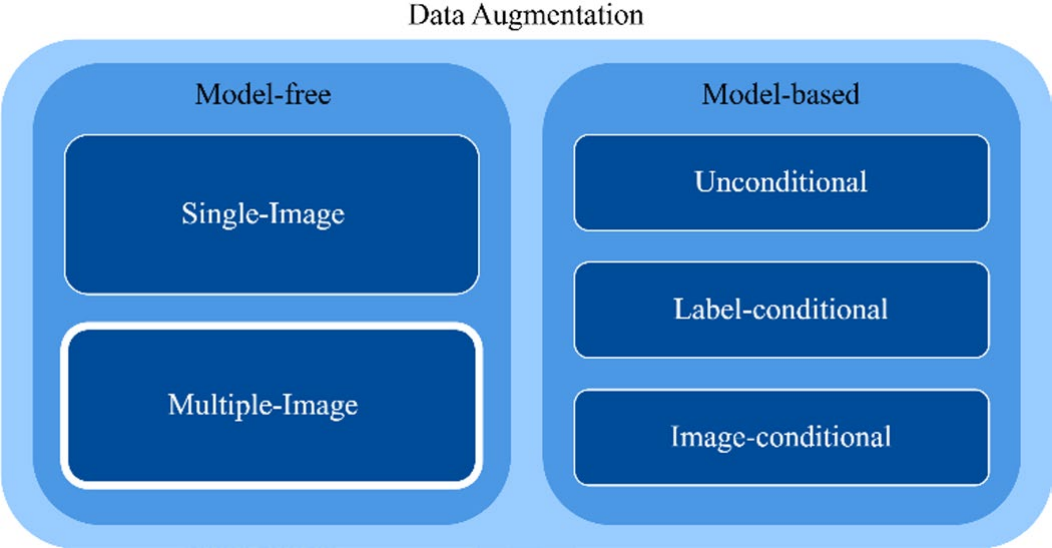
Data augmentation taxonomy.

Data augmentation is a critical technique for DL, particularly in fields such as GI endoscopy, where datasets are often small, imbalanced, and lack diversity. It generates new, diverse samples, helping to reduce overfitting, improve model robustness, and enhance performance in detecting rare or underrepresented lesions. Augmentation methods can be mainly categorized into model-free and model-based approaches^7–9^(Fig. 1).

Model-free methods do not rely on external models and include single-image transformations, such as rotations, flips, and intensity adjustments, which introduce variability while preserving the original image content. Additionally, multiple-image techniques, such as Mixup^10^ and CutMix^11^, combine features from different images to create richer, hybrid samples that balance class representation and improve dataset diversity^7,8^.

Model-based methods utilize generative models to synthesize new data. These include unconditional methods (e.g., DCGAN12), label-conditional methods (e.g., BAGAN13), and image-conditional approaches (e.g., AugGAN14). Model-based approaches (including more recent diffusion- and transformer-based generators) can provide powerful sample diversity but often require large training sets and careful validation to ensure clinical realism. ^7,8^.

Beyond generative approaches, recent work has also explored mathematically structured or transformation-driven augmentation frameworks. For example, Selvaraj and Jayanthly proposed a principled augmentation methodology designed to improve classifier robustness under limited or imbalanced training conditions. While such methods contribute to the broader augmentation landscape, they operate at the image-transformation level and do not address the instance-level, anatomy-aware synthesis required for subtle GI lesions^12^.

In this study we exclude policy-optimisation augmentation schemes, as these focus on selecting or scheduling augmentations rather than on generating novel lesion-bearing samples.

### Multiple image combination methods

**Fig. 2:**
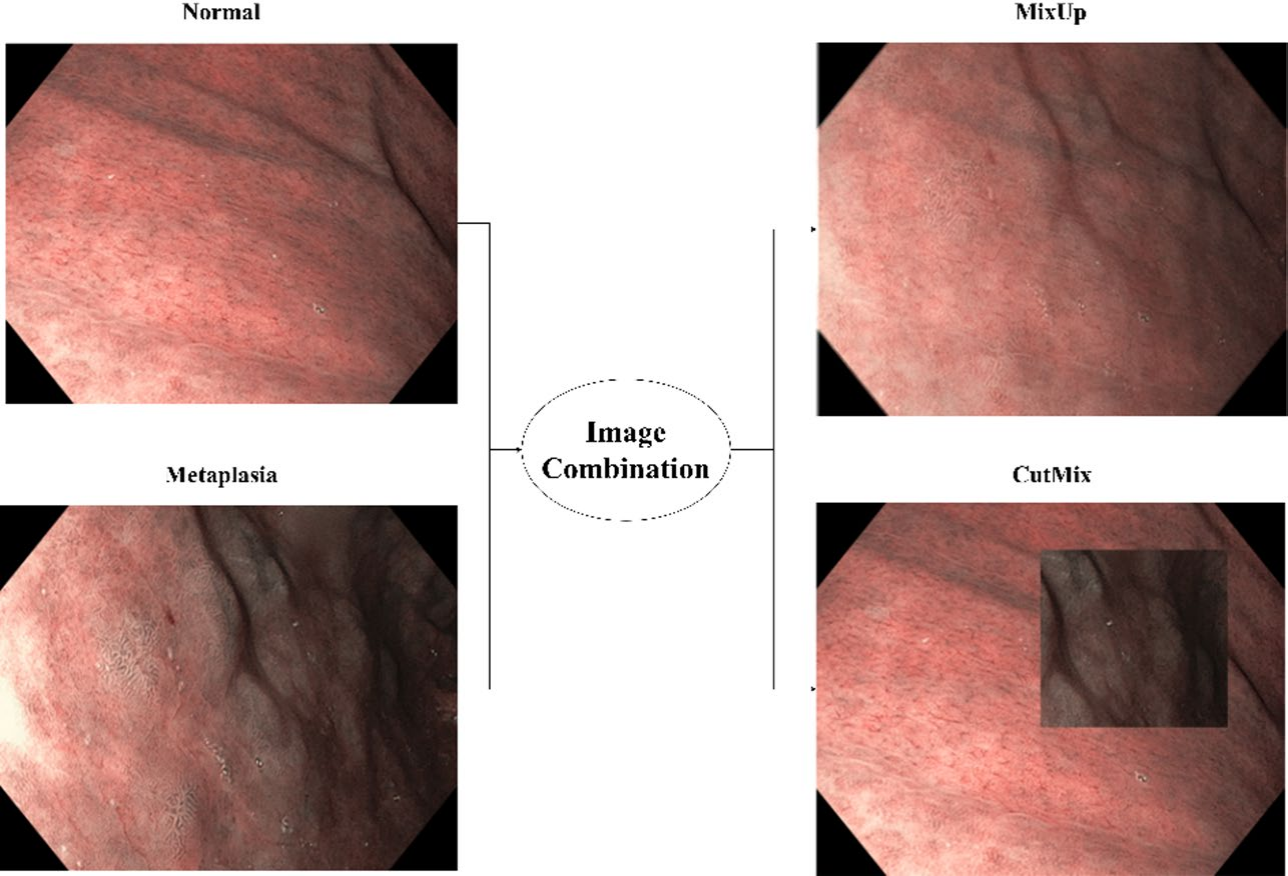
Examples of MixUp and CutMix output for the IM domain context.

Image-combination techniques generate new training samples by combining parts from multiple images, normally from two different classes. Two widely used methods in this category are MixUp and CutMix (Fig. 2):

**MixUp** – combines two images by performing a linear interpolation between their pixel values and blending their corresponding labels. For instance, given two images *x*_*i*_ and *x*_*j*_ with labels *y*_*i*_ and *y*_*j*_, a new sample is created as:

1 $${x}_{new}=\lambda{x}_{i}+\left(1-\lambda\right){x}_{j}$$

2 $${y}_{new}=\lambda{y}_{i}+(1-\lambda){y}_{j}$$

Here, the λ is a mixing coefficient sampled from a Beta distribution. This method generates smooth transitions between classes and encourages the model to learn more generalized and robust decision boundaries. MixUp is particularly effective for datasets with noisy labels or highly overlapping class distributions, as it smoothens out overfitting tendencies by exposing the model to mixed examples^10^.

**CutMix** – takes a different approach by cutting a rectangular patch from one image and pasting it onto another image. The labels are also mixed proportionally based on the area of the patch. Formally, given two images xi and xj with labels yi and yj, the new sample is created as:

3 $${x_{new}} = {x_i} \odot M + {x_j} \odot (1 - M)$$

4 $${y}_{new}=\lambda{y}_{i}+(1-\lambda){y}_{j}$$

Where M is a binary mask that defines the rectangular region, ⨀ is an element-wise multiplication, and λ corresponds to the area of the cut patch relative to the whole image. Unlike MixUp, CutMix retains spatial integrity in the untouched regions of the images, allowing the model to learn localized features while still benefiting from augmented diversity^11^.

Both techniques aim to improve model generalization by exposing it to a broader distribution of training samples. MixUp is more suited for scenarios requiring smooth label transitions, while CutMix excels in tasks where preserving spatial structure is critical for learning distinct features^8–11^.

### Related studies

Data augmentation plays a vital role in medical imaging, especially for training DL models with limited and imbalanced datasets. In the early stages, general methods such as MixUp^10^ and CutMix^11^ have become essential tools for improving model generalization. While these methods enhance model robustness, they are less effective for fine-grained tasks like GI lesion detection due to their lack of consideration for feature localization^13^.

While MixUp blends entire images, helping with regularization, it tends to obscure fine-grained details like lesion boundaries, which are essential for detecting subtle abnormalities. CutMix attempts to preserve spatial integrity by pasting patches from one image into another. However, its random patch placement can dilute important features, making it less effective for tasks requiring precise localization, such as GI lesion detection^7–9,13^.

Recent efforts have attempted to address this challenge. For instance, Gradient Saliency-aware CutMix^14^ leverages saliency maps to guide patch placement, ensuring that key features such as lesions are preserved during augmentation. This adaptation improves performance but adds complexity by requiring external saliency models, which may introduce variability and reduce model robustness.

### From non-instance-level to instance-level augmentation

**Fig. 3:**
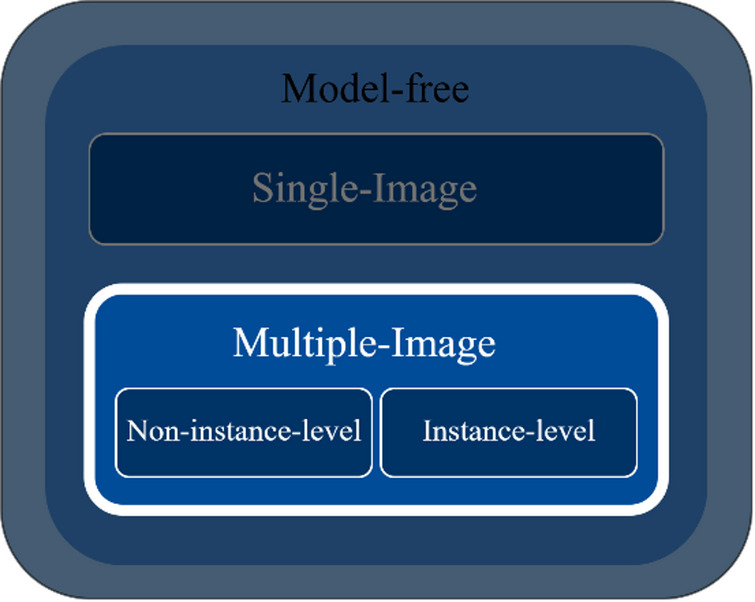
Types of multiple-image methods: non-instance and instance level.

As the need for more specialized augmentations grew, cut-and-paste methods emerged as an effective solution. These techniques focus on copying specific regions of interest from one image and embedding them into another. Unlike generic augmentation methods like MixUp and CutMix (non-instance-level), cut-and-paste methods allow for instance-level augmentation, offering more control over the features preserved during augmentation^7^(Fig. 3).

CycleMix^15^, for example, demonstrates a holistic strategy where lesions or objects from one image are transferred to another. This approach is particularly effective for tasks involving segmentation and classification when annotations are sparse or incomplete. Balanced-MixUp^16^ introduces an improvement by incorporating balancing mechanisms that enhance the performance of highly imbalanced datasets, common in medical imaging, such as when certain lesion types are underrepresented. By maintaining balanced classes during augmentation, these methods further increase the model’s ability to generalize and detect rare lesions.

Copy-paste augmentation has also proven effective in object detection. Studies like Simple Copy-Paste^17^ and Cut, Paste, and Learn^18^ have demonstrated the success of straightforward cut-and-paste techniques for instance segmentation. These methods have been found to significantly boost model performance by preserving specific object features while enhancing diversity within the training set. While these advancements have not been directly applied to medical imaging, their simplicity and effectiveness present clear potential for adaptation in this field. Modelling Visual Context^19^ emphasizes the importance of considering the surrounding context of pasted regions to maintain biological relevance. This is a crucial factor for medical tasks, where the spatial relationship between lesions and other anatomical structures can significantly affect diagnosis.

### Biomedical adaptations

The transition from generic cut-and-paste methods to domain-specific adaptations in biomedical imaging has led to the development of techniques that address the unique challenges of localized and irregular lesions. These adaptations often focus on anatomical plausibility and the preservation of biologically relevant features.

LesionMix^20^ represents a key development in this area. It performs lesion-level augmentation by copying annotated lesion regions and embedding them into different image contexts. This approach maintains anatomical plausibility, making it particularly effective for segmentation tasks in medical imaging. However, its effectiveness can be influenced by how well the pasted lesions integrate into their new backgrounds, as differences in texture and illumination may create inconsistencies that require additional post-processing to correct.

Building on this concept, Soft-CP^21^ improves upon the basic cut-and-paste technique by blending lesions into target images with smooth transitions. By employing gradient-based blending, Soft-CP ensures that the augmented samples are realistic and preserve the subtle boundaries of lesions, which is essential for tasks such as polyp detection in GI imaging. This method addresses some of the limitations of LesionMix by ensuring smooth integration of lesion features, thus increasing realism and preserving diagnostic quality.

Another significant contribution is TumorCP^22^, which adapts the cut-and-paste paradigm for tumour segmentation. Similar to LesionMix, TumorCP enhances dataset diversity by copying and pasting tumour regions into different contexts. However, its focus on increasing the variability of tumour appearances makes it particularly useful for tumour detection across diverse imaging scenarios. These methods share similar principles with LesionMix, yet they demonstrate varying levels of complexity and adaptability depending on the type of lesions or tumours involved.

### Towards domain-specific augmentation for gastrointestinal imaging

GI imaging presents unique challenges due to irregular lesion morphology, subtle boundaries, and strong dependence on the surrounding mucosal context. While multiple-image augmentation approaches (e.g., MixUp, CutMix) and recent copy–paste adaptations have demonstrated value in natural and biomedical images, their direct application to GI lesions remains limited by anatomical constraints and domain-specific characteristics.

### Limitations of existing approaches

Based on the approaches reviewed, existing augmentation strategies present three main recurrent limitations when applied to GI endoscopic images:

(A) Non-instance-level mixing (MixUp, CutMix).

- **MixUp dilutes local lesion structure**, blending pixel intensities across entire images and obscuring subtle boundaries essential for detecting early IM or dysplasia.
- **CutMix inserts rectangular patches without anatomical awareness**, ignoring mucosal continuity; pasted regions frequently violate GI anatomy or appear in implausible locations.

(B) Instance-level copy–paste methods (LesionMix, Soft-CP, TumorCP, Simple Copy-Paste).

Instance-level augmentation techniques represent a valuable evolution beyond global mixing strategies, but they remain limited when applied to GI endoscopy. Biomedical variants such as LesionMix and Soft-CP focus on transferring lesion patches and smoothing their boundaries. Yet, they do not enforce anatomical or visual compatibility between the pasted lesion and the target mucosal region. Because GI tissue varies substantially in texture, colour and illumination, this lack of contextual modelling may lead to inconsistencies in augmented samples.

Similarly, copy–paste methods originally developed for natural images assume clearly defined object contours and do not incorporate any form of mucosal structure modelling or region-similarity criteria, making them suboptimal for subtle and irregular GI lesions. Approaches like TumorCP, while effective for large, well-delimited tumours, do not target the fine-grained, low-contrast patterns typical of IM and dysplasia.

(C) Model-based generative approaches (GANs, AugGAN).

- Require **large training datasets** not available for GI lesions.
- Can introduce **hallucinated artefacts** that compromise clinical reliability.
- Provide **little control** over lesion placement, or boundary morphology, or presence of relevant class features.

These limitations highlight the need for an augmentation strategy that jointly ensures anatomical plausibility, contextual compatibility and preservation of fine-grained lesion morphology.

### Motivation

Given the scarcity of annotated GI datasets and the limitations of non-contextual or anatomically unaware augmentation approaches, there is a need for a domain-specific method that:

- generates realistic, anatomically coherent synthetic GI lesions,
- preserves subtle lesion boundaries and local mucosal patterns,
- reduces class imbalance for less represented lesion types,
- improves cross-dataset generalisation,
- and scales effectively without requiring generative modelling or external saliency networks.

### Objectives

This study aims to:

#### O1

Develop an instance-level augmentation method that selects biologically plausible target regions using unsupervised region proposal and appearance similarity.

#### O2

Achieve seamless integration of lesions through Poisson image editing combined with gradient-based blending.

#### O3

Evaluate the method across IM, dysplasia and polyp datasets using 5-fold cross-validation and external testing.

#### O4

Compare performance with MixUp, CutMix and naive copy–paste augmentation using classification and Grad-CAM-based metrics.

#### O5

Assess the influence of blending parameter α on performance stability.

#### O6

Provide implementation details and discuss limitations and future directions.

### Contributions

The contributions of this work are:

#### C1

We propose **Cut Instance Mixing (CIM)**, a domain-specific augmentation method that integrates unsupervised region proposal (MeanShift + SLIC), appearance-based matching, Poisson editing and gradient blending.

#### C2

CIM generates anatomically coherent, labelled synthetic samples that preserve fine-grained lesion features and mitigate class imbalance.

#### C3

We conduct extensive evaluation across three GI lesion datasets, including external testing, showing improved classification performance and increased alignment between Grad-CAM maps and true lesion regions.

#### C4

CIM is lightweight, scalable, and avoids reliance on generative models or externally trained saliency networks, enabling deployment in data-scarce clinical settings.

### Methodology

In this work, we propose Cut Instance Mixing (CIM), a domain-specific data augmentation method designed to address the limitations of state-of-the-art image combination techniques, such as MixUp and CutMix, when applied to GI endoscopy images. To evaluate the effectiveness of our approach, we use three distinct datasets, each corresponding to a specific type of GI lesion: IM, dysplasia, and polyps.

The experimental pipeline involves generating augmented datasets using MixUp, CutMix, and the proposed CIM methods. These data augmentation methods are subsequently used to train a ResNet50 model for lesion detection tasks. The performance and generalisation capability of the trained models are then evaluated and compared across all augmentation methods.

**Fig. 4:**
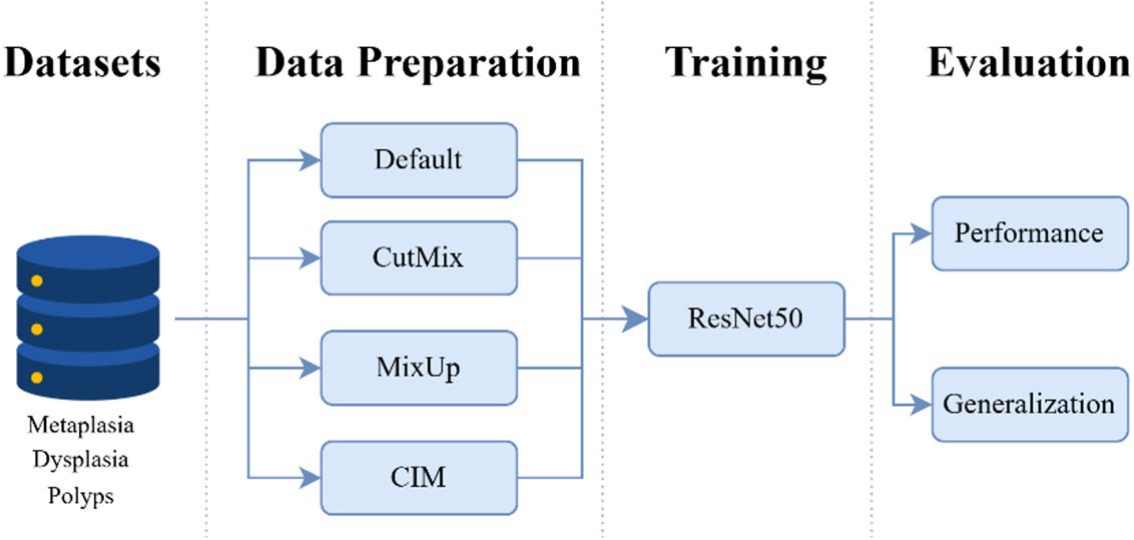
General overview of the global pipeline for this work.

The overall workflow is summarized in Fig. 4, which illustrates the data flow, augmentation strategies, and evaluation metrics used in our study.

### Datasets

**Fig. 5:**
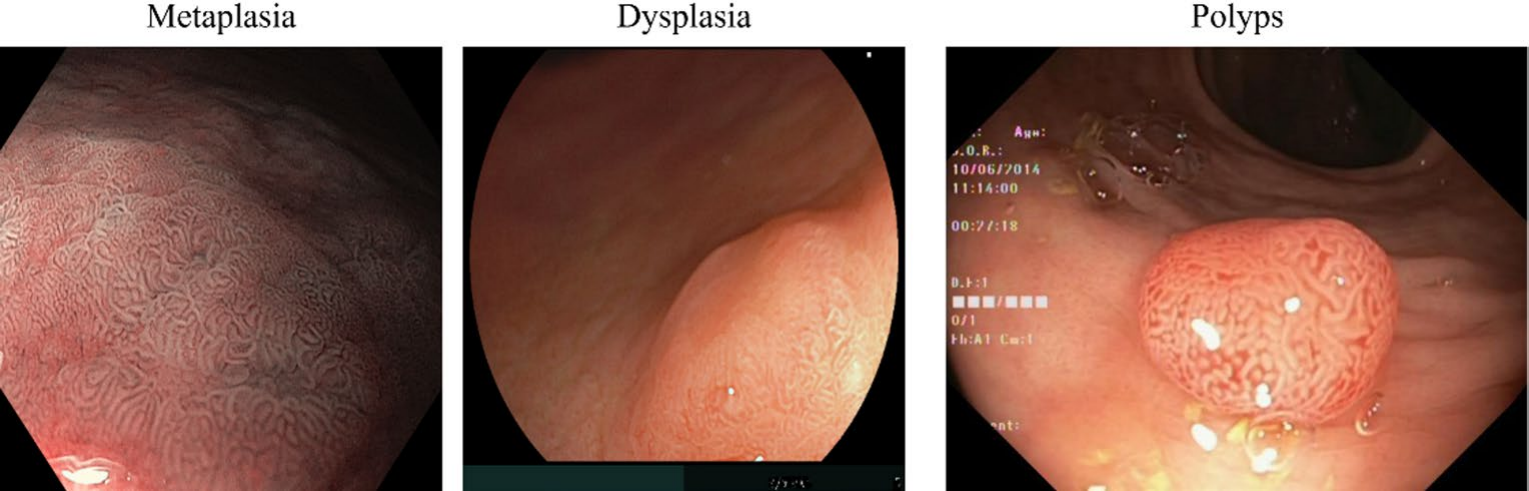
Examples of images used for this work, regarding IM, dysplasia and polyps.

For this study, we selected three distinct GI lesions: IM and dysplasia from the upper GI tract, and polyps from the lower tract (Fig. 5).

- **Metaplasia Dataset**: Dataset collected on regular clinical practice at the Instituto Português de Oncologia do Porto (IPO-Porto, Portugal), comprising a total of 690 narrow-band imaging images (523 Normal and 167 IM). All these images were collected using an OLYMPUS CV-190 endoscope and present a 24-bit colour depth. Additionally, these images include two different resolutions: 475 images (410 Normal and 65 IM) of high-resolution (1350 × 1080 pixels) and 215 images (113 Normal and 102 IM) of medium resolution (640 × 480 pixels).
- **Dysplasia Dataset**: Similarly, this dataset was also gathered at IPO-Porto on regular clinical practice and includes 370 white light endoscopy images (370 Normal and 98 dysplasia/early cancer) with 24-bit colour depth and 1280 × 1024 pixels resolution. All these images were collected using a FUJIFILM VP-7000 endoscope.
- **Polyps Dataset**: This dataset comprises 200 normal and 32 lesion images for training a ResNet50 model, with an external test set of 496 normal and 306 lesion images. Images were collected under varying conditions from multiple hospitals and endoscopic devices to ensure diversity. It integrates data with resolutions ranging from 332 × 487 to 1920 × 1072 pixels and a 24-bit colour depth^23–25^.

This combination of datasets enables a comprehensive assessment of the proposed method’s performance, specifically within the unique challenges of endoscopic imaging. Variations in lighting, image modality, noise, and the presence of different tissue types can impact model performance, making it essential to evaluate CIM across structurally and visually distinct regions of the GI tract. Additionally, the inclusion of an external test set from different hospitals and endoscopic devices ensures a robust evaluation of CIM’s generalization to diverse imaging conditions, reinforcing its clinical applicability. All patients were informed about the research purposes and signed clarified written consents, and the Ethical Committee of IPO-Porto approved the data usage for this study (Parecer CES. 154/023). All methods were performed in accordance with the relevant guidelines and regulations.

### Data preparation

A 5-fold cross-validation strategy was applied independently to each dataset after an 80/20 stratified split into training and test sets. Data augmentation was performed exclusively on the training portion, where synthetic lesion samples were generated in a 1:1 ratio with respect to the number of normal training images, ensuring balanced class distributions. Each augmented dataset variant (MixUp, CutMix, simple copy–paste, CIM without blending, and CIM with α ∈ {0, 0.2, 0.4, 0.6, 0.8, 1}) was created following the same protocol. All images were resized to 224 × 224 before training.

Table 1 summarises the resulting number of real and synthetic samples per class, the per-fold composition under 5-fold cross-validation, and the corresponding test and external test sets.

**Table 1:** Databases split information.

	Class	Real train	Aug. Train	Test	Per fold (Real/Aug.)	Total	External Test
IM	Lesion	134	418	33	≈ 33 real/105 aug.	167	-
	Normal	418	-	105	105	523	-
Dysplasia	Lesion	79	296	19	≈ 19 real/74 aug.	98	-
	Normal	296	-	74	74	370	-
Polyps	Lesion	26	160	6	≈ 6 real/40 aug.	32	306
	Normal	160	-	40	40	200	496

### Cut instance mixing – CIM

**Fig. 6:**
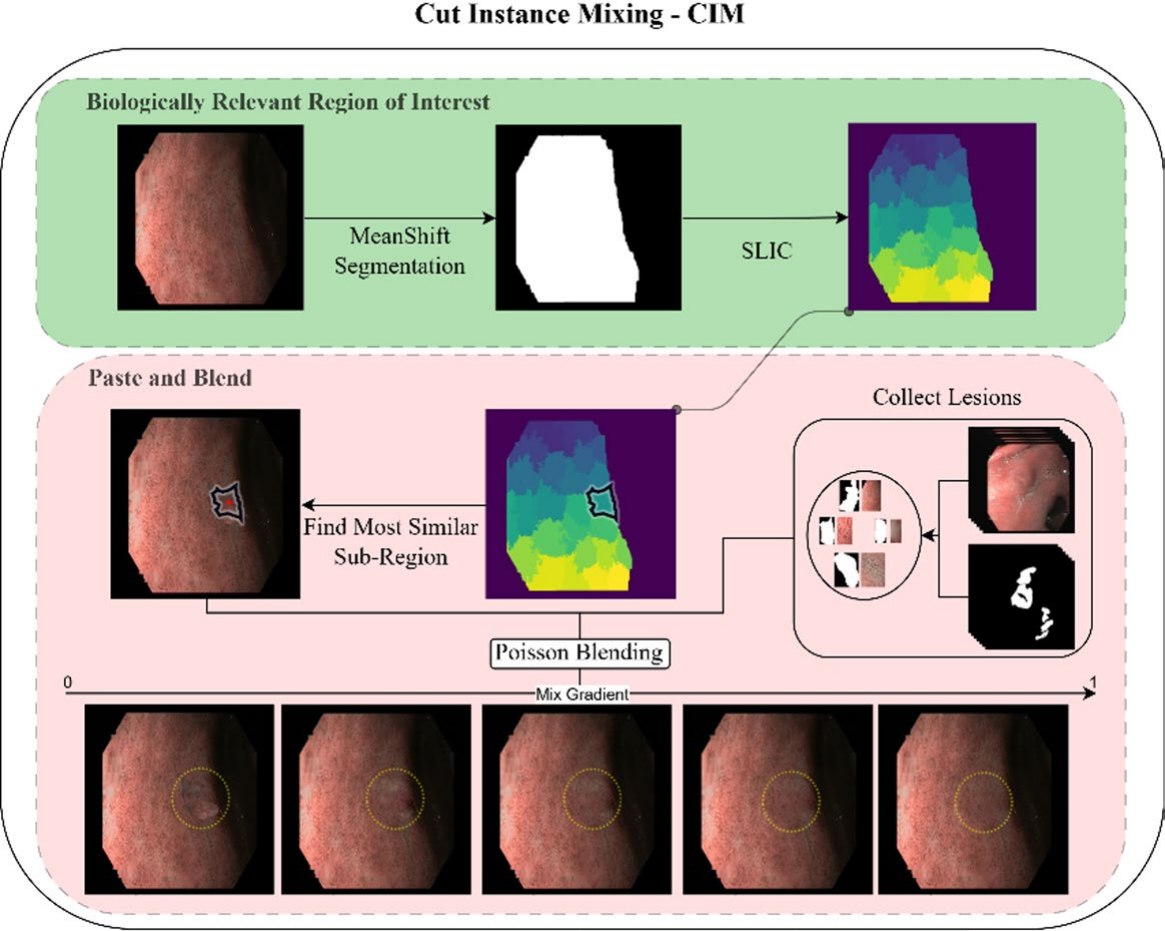
Cut Instance Mixing – CIM. **Green** – Biologically Relevant Region of Interest detection. **Red** – Past and Blending of lesion instances.

The CIM method improves traditional image combination techniques through a two-main-step process (Fig. 6):

**(1)** identification of biologically relevant regions of interest (ROIs) in normal images.

**(2)** pasting and blending lesion instance features into these regions.

This approach motivates the creation of realistic lesion-containing samples that better mimic endoscopic conditions. Additionally, the method is versatile and can be adapted to other types of images, where relevant ROIs are identified, and the feature of interest is then pasted and blended.

### Biologically relevant region of interest

The process begins by applying a Mean Shift algorithm^26^ to identify clusters within the image. This non-parametric method iteratively shifts pixels towards the densest areas of feature space, using both colour and spatial information. It groups pixels into homogeneous regions, which are then selected based on their biological relevance to the given problem. This allows for the identification of regions that are biologically significant, such as potential areas of interest in medical images. After this, a simple linear iterative clustering (SLIC) method^27^ is employed to further divide the image into smaller sub-regions. SLIC is a superpixel segmentation technique that groups pixels into compact, uniform regions based on both colour and spatial proximity. The algorithm assigns pixels to cluster centres, refining them iteratively to form coherent, manageable sub-regions. These sub-regions are used as candidate locations for pasting lesion features, ensuring they align with the biological context of the image.

### Paste and blend

After applying the SLIC method to segment the image into candidate sub-regions, a random lesion instance is selected. To ensure optimal blending, the most similar sub-region is identified by comparing its properties—such as texture and colour—to those of the lesion. The mean colour of each superpixel is computed, and the Euclidean distance between the lesion’s mean colour and each candidate superpixel is calculated. The sub-region with the smallest distance is then chosen as the best match for seamless integration. The lesion may undergo geometric augmentation (e.g., flipping and rotation) to ensure variation. It is then pasted at the centre of the selected sub-region. To seamlessly integrate the lesion with the background, a Poisson image editing method is applied. This method preserves gradient continuity, ensuring smooth transitions and consistent lighting while minimizing edge artefacts for a natural blend^28^. Let the lesion image be denoted by L, the background by B, and the final blended image by I. The Poisson blending is formulated as:

5 $${\nabla}I\left(x\right)={\nabla}B\left(x\right)+{\nabla}L\left(x\right),x\in{\Omega}$$

Where ∇ represents the image gradient, and Ω is the region where the lesion is placed. This equation ensures that the gradient of the blended image *I* matches the gradients of the background *B* and the lesion *L*.

Additionally, to control the blending effect, a gradient mixing function with the α parameter is applied. The blending between the lesion and the background can be adjusted by varying α:

6 $$I\left(x\right)={\alpha}L\left(x\right)+\left(1-\alpha\right)B\left(x\right)$$

Where:

- α ∈ [0, 1] controls the balance between the lesion and the background.
- Lower α values emphasize the lesion, while higher values prioritize blending with the background.

This approach ensures that the lesion blends naturally with its surroundings, making the augmented samples more realistic for training DL models.

### Training

The ResNet50 model^29^, pre-trained on ImageNet, was used as the base, with a global average pooling layer followed by two dense layers (1024, 512 units) and a dropout rate of 0.3. The Adam optimizer with a learning rate of 1 × 10^−4^ was used, along with a binary focal loss function. Training included early stopping after 10 epochs without improvement and learning rate reduction after 3 epochs of no progress. The model was trained for 100 epochs.

**Fig. 7:**
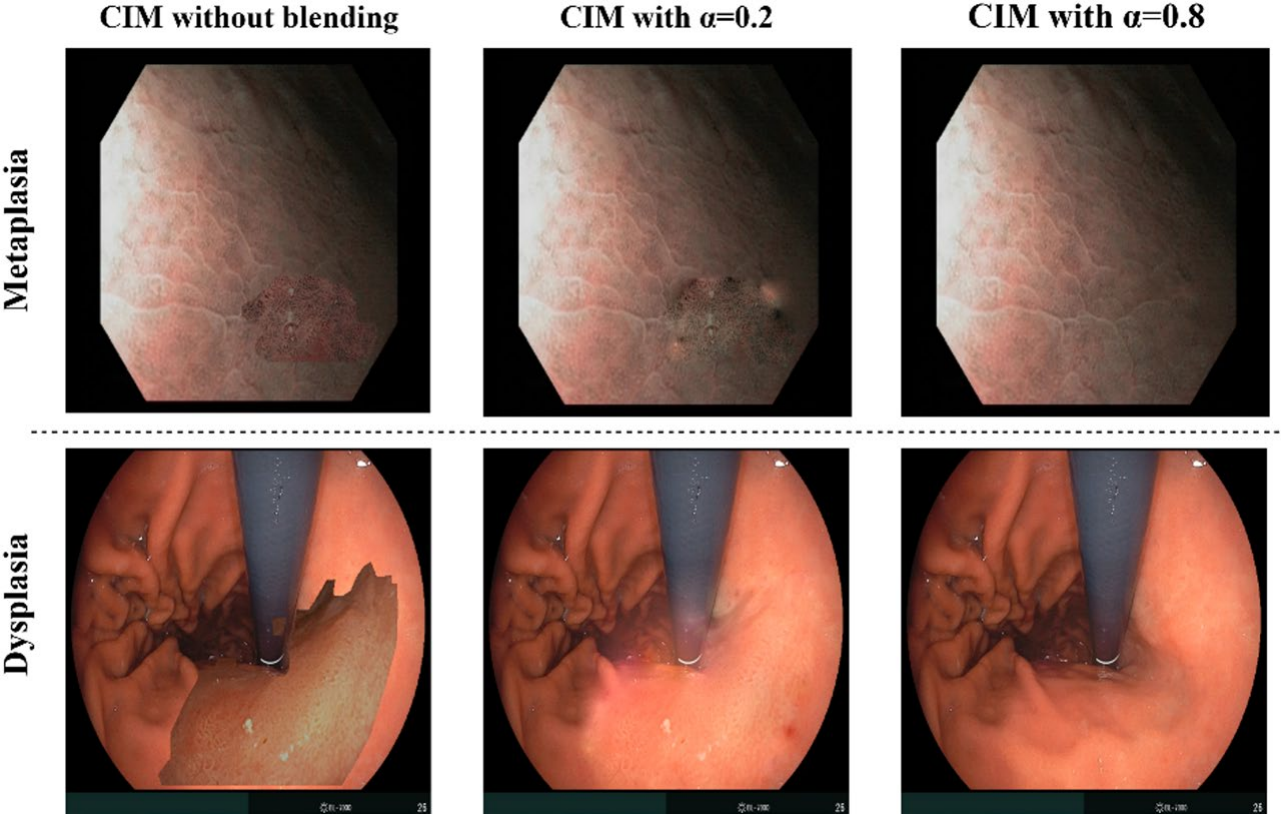
Examples of synthetic IM and dysplasia images from the CIM method. CIM method without lesion blending in the first column and CIM with a mixed gradient at α = 0.2 and 0.8.

Figure 7. shows some examples of augmented images created by our approach – the CIM method.

The training was conducted using the following data augmentation methods:

- Default;
- CutMix;
- MixUp;
- Lesions randomly pasted into images;
- CIM without blending;
- CIM with varying α mix gradients degrees (0 to 1, in steps of 0.2).

### Evaluation

To evaluate the model’s performance and generalization capabilities, we used the IM and dysplasia datasets to assess the model’s performance on the same datasets used during training. This allows us to evaluate how well the models perform within the specific context of the data they were trained on. Additionally, the polyp classification model was tested on an external test set collected from different hospitals, under varying conditions, to evaluate the model’s generalisation ability. This approach provides insights into how well the models can adapt to new, unseen data, ensuring their robustness in real-world clinical scenarios.

All results will be evaluated using Repeated Measures ANOVA to identify significant differences across methods for each metric. For any significant findings, post hoc analyses will be performed, incorporating statistical measures such as Hedges’ g to determine effect sizes, Bayes Factors (BF10) for evidence strength, and adjusted p-values to control for multiple comparisons. This comprehensive statistical approach ensures a rigorous and nuanced interpretation of the performance results.

## Results

In this section, we present the evaluation of our model across two key aspects: general performance and generalisation capabilities. For general performance, we analyze the model’s results on the IM and dysplasia datasets. To assess generalisation capabilities, we train the model using the polyp dataset and evaluate it on an external test set. This approach allows us to investigate the model’s ability to adapt to unseen data while maintaining robust performance.

### General performance

**Fig. 8:**
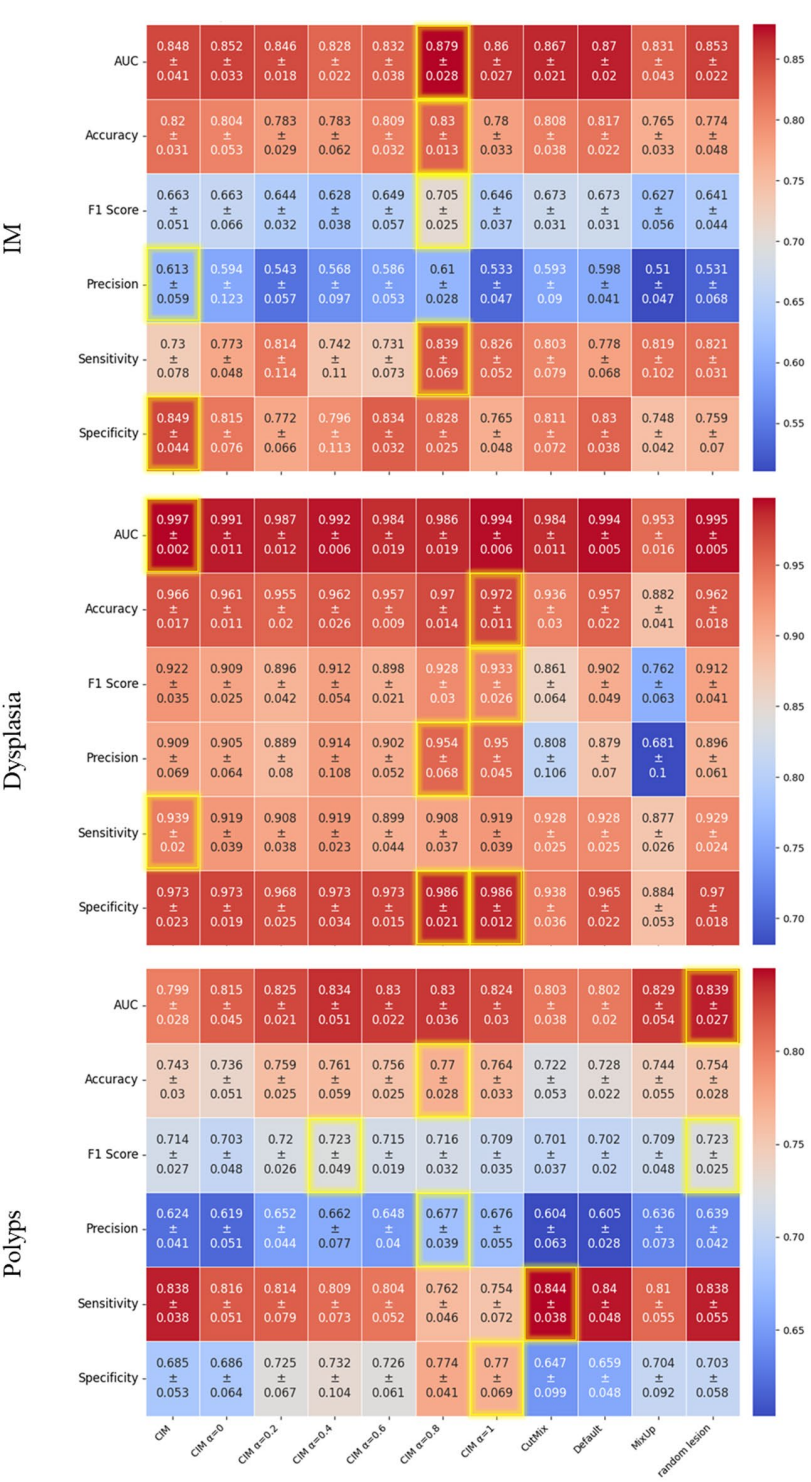
Mean results and standard deviation for each experiment, with 5-fold cross-validation, regarding the IM, dysplasia and polyps’ classification.

The results on the IM dataset reveal that CIM with α = 0.8 consistently outperforms other methods across multiple metrics. The AUC achieves the highest value of 0.879 ± 0.028, surpassing both MixUp (0.765 ± 0.037) and CutMix (0.867 ± 0.027), while the default approach lags behind at 0.817 ± 0.031. Similarly, Accuracy peaks at 0.823 ± 0.037 with CIM (α = 0.8), outperforming MixUp (0.765 ± 0.037) and CutMix (0.808 ± 0.038). The F1 Score and Precision follow the same trend, with CIM (α = 0.8) achieving the highest values (0.705 ± 0.025 and 0.661 ± 0.025, respectively), while MixUp and CIM with α = 0.4 yield noticeably lower scores. Sensitivity is also highest for CIM (α = 0.8) at 0.839 ± 0.052, whereas the default approach and MixUp perform comparably lower. Lastly, Specificity remains strong across methods, with CIM achieving the top value of 0.849 ± 0.044 (Fig. 8).

The results on the IM dataset reveal that CIM with α = 0.8 consistently outperforms other methods across multiple metrics. The AUC achieves the highest value of 0.879 ± 0.028, surpassing both MixUp (0.765 ± 0.037) and CutMix (0.867 ± 0.027), while the default approach lags behind at 0.817 ± 0.031. Similarly, Accuracy peaks at 0.823 ± 0.037 with CIM (α = 0.8), outperforming MixUp (0.765 ± 0.037) and CutMix (0.808 ± 0.038). The F1 Score and Precision follow the same trend, with CIM (α = 0.8) achieving the highest values (0.705 ± 0.025 and 0.661 ± 0.025, respectively), while MixUp and CIM with α = 0.4 yield noticeably lower scores. Sensitivity is also highest for CIM (α = 0.8) at 0.839 ± 0.052, whereas the default approach and MixUp perform comparably lower. Lastly, Specificity remains strong across methods, with CIM achieving the top value of 0.849 ± 0.044 (Fig. 8).

All methods demonstrate strong performance on the dysplasia dataset, but CIM with α = 0.8 and α = 1 consistently achieve superior results. The AUC reaches near-perfect values of 0.997 ± 0.002 for both CIM (α = 0.8) and CIM (α = 1), clearly outperforming MixUp (0.953 ± 0.009) and random lesion pasting (0.955 ± 0.008). Accuracy peaks at 0.966 ± 0.011 for CIM, outperforming MixUp (0.882 ± 0.017) and random lesion pasting (0.903 ± 0.012). Similarly, the F1 Score and Precision are highest for CIM (α = 0.8 and α = 1), while MixUp and random lesion pasting exhibit noticeably lower values. Sensitivity reaches an almost perfect 0.999 ± 0.001 for CIM, far exceeding MixUp (0.877 ± 0.020). Specificity also shows strong results, with CIM (α = 0.8) achieving the highest score of 0.986 ± 0.012, surpassing MixUp (0.759 ± 0.047) and the default approach (Fig. 8).

In summary, CIM with α = 0.8 and α = 1 consistently achieves the best overall performance on both datasets, outperforming state-of-the-art methods such as MixUp and CutMix, as well as the default random copy-paste lesion approach. This demonstrates the effectiveness of the proposed method, particularly in challenging tasks like IM detection.

#### Generalization capabilities

The results for the polyp dataset, used to assess generalization capabilities, show that the proposed CIM strategies outperform the default method and state-of-the-art approaches like MixUp and CutMix across key metrics (Fig. 8). CIM with α = 0.8 achieves the highest Accuracy (0.769 ± 0.028) and Specificity (0.774 ± 0.041), clearly improving over the default method (0.728 ± 0.022) and MixUp (0.744 ± 0.055). CIM with α = 0.4 and α = 1 also perform competitively.

For Precision and F1 Score, CIM strategies, particularly with α = 0.4 and α = 0.8, consistently achieve better results than the default, MixUp, and CutMix.The AUC is highest for CIM with α = 0.8 (0.830 ± 0.036) and CIM with α = 0.6, outperforming all other methods, including MixUp and CutMix.

In summary, CIM strategies, especially with α = 0.8, demonstrate superior generalization capabilities, achieving the best overall performance across key metrics compared to state-of-the-art methods and the default approach.

## Discussion

### General performance

**Fig. 9:**
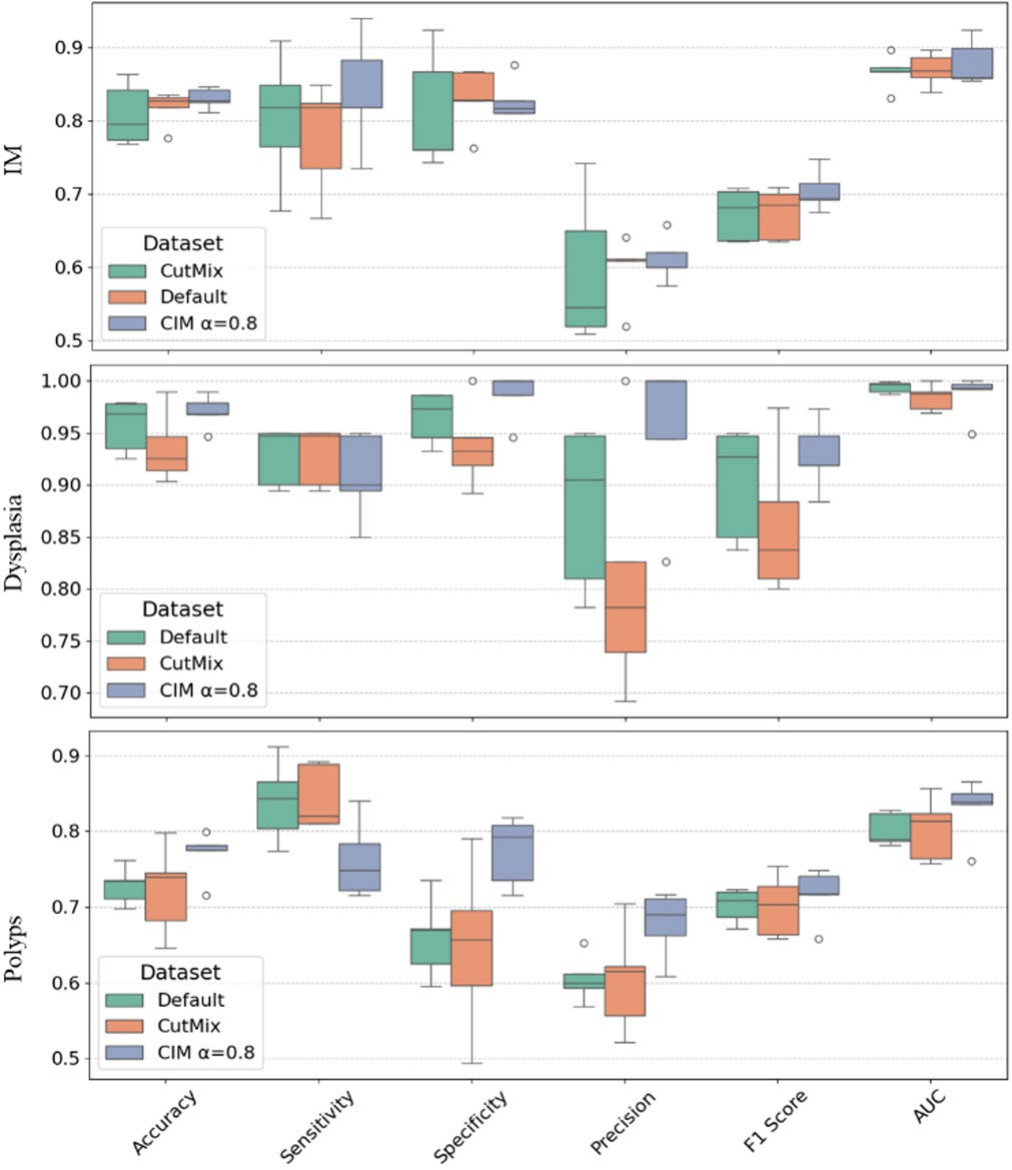
Boxplots for the IM, dysplasia and polyps’ classification.

In the general performance evaluation for IM detection, CIM with α = 0.8 consistently achieves the best results, showing both higher mean values and lower variability across folds, as evidenced by the boxplots (Fig. 9). This combination highlights the method’s robustness and reliability when compared to state-of-the-art approaches like CutMix and MixUp.

For the IM dataset, CIM with α = 0.8 excels in most metrics, achieving the highest AUC (0.879 ± 0.028), Accuracy (0.823 ± 0.037), and F1 Score (0.705 ± 0.025). It also delivers strong Sensitivity (0.839 ± 0.052), indicating its effectiveness at identifying true positives. However, its Specificity slightly lags behind the default method, suggesting room for improvement in reducing false positives. Compared to Default and CutMix, CIM shows both higher performance and lower variability, while these methods display greater instability across folds.

The analysis began with a Repeated Measures ANOVA, which showed a significant difference in F1 Score (*p* = 0.0328) but no significant differences in Accuracy, Sensitivity, Specificity, or Precision. Post-hoc pairwise comparisons revealed that CIM with α = 0.8 significantly outperformed the Default method in F1 Score (*p* = 0.03598), with a Bayes Factor (BF10) of 2.806 indicating moderate evidence for CIM’s superiority. The Hedges’ g value of 0.901 suggests a moderate effect size.

When comparing CIM α = 0.8 to CutMix, no significant difference was found (*p* = 0.0939). The BF10 of 1.422 suggests weak evidence in favour of CIM, with a Hedges’ g of 0.912 showing a moderate effect size, but the difference did not reach statistical significance after FDR correction (*p* = 0.1409). This suggests CIM performs slightly better than CutMix, but the evidence is not strong enough to confirm a definitive advantage.

In dysplasia detection, CIM with α = 1 again dominates, reaching near-perfect AUC (0.994 ± 0.006), with similarly strong Accuracy, F1 Score, and Sensitivity. In contrast, CutMix and Default show higher variability, particularly in Precision and Specificity, limiting their overall reliability despite some competitive results (Fig. 9).

A Repeated Measures ANOVA revealed no significant differences in Accuracy, Sensitivity, F1 Score, or AUC across methods. However, significant differences were observed for Specificity (*p* = 0.0439) and Precision (*p* = 0.0406), highlighting variability in the ability of the methods to minimize false positives and maintain precision.

Post-hoc pairwise comparisons for Specificity showed that CIM α = 1 tended to outperform CutMix (*p* = 0.0533, BF10 = 2.119, Hedges’ g = 1.469) and Default (*p* = 0.0777, BF10 = 1.624, Hedges’ g = 0.986), although these results did not reach statistical significance. CutMix and Default were not significantly different (*p* = 0.2204). Similarly, for Precision, CIM α = 1 demonstrated better performance than CutMix (*p* = 0.0513, BF10 = 2.178, Hedges’ g = 1.412) and Default (*p* = 0.0667, BF10 = 1.809, Hedges’ g = 0.979). Again, CutMix and Default showed no significant difference (*p* = 0.2432).

**Fig. 10:**
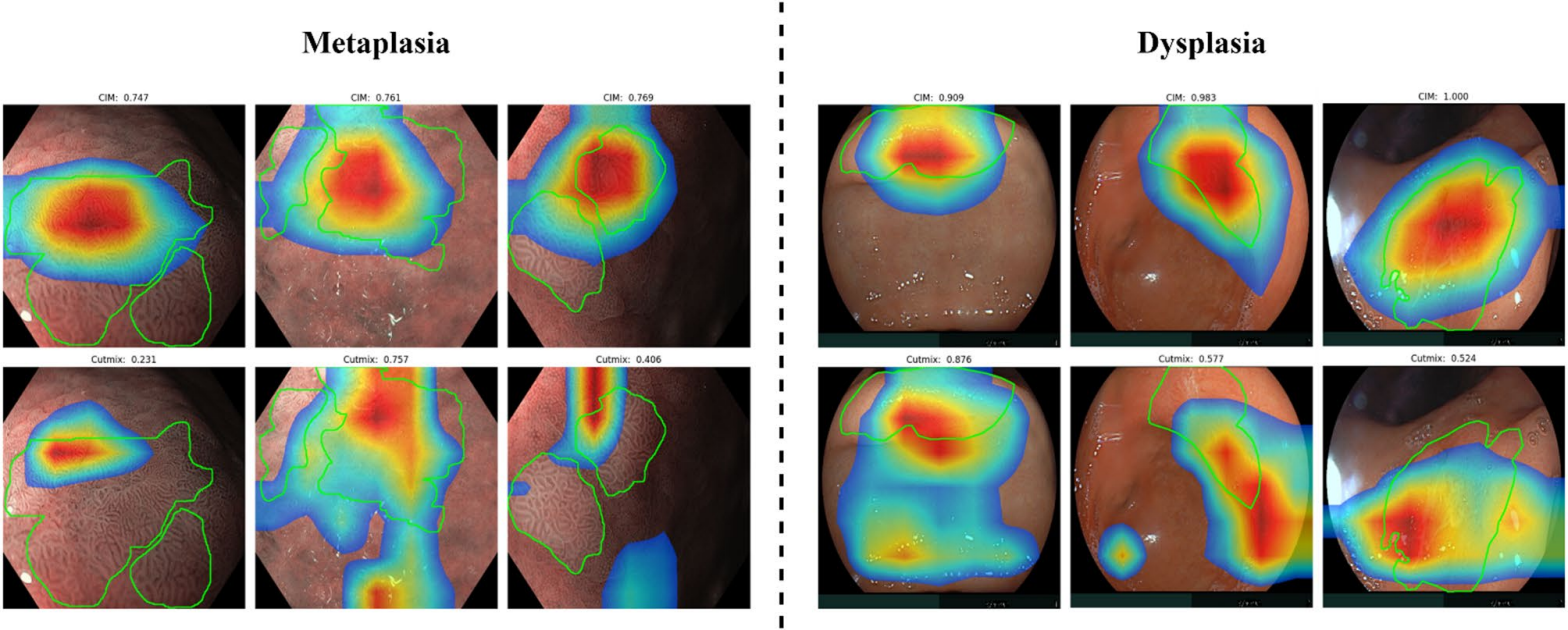
Grad-Cam heatmap examples for IM and dysplasia endoscopy image classification.

These results suggest that while the overall differences were not statistically significant, CIM α = 1 displayed consistently better performance in minimizing false positives (Specificity) and maintaining consistent Precision compared to CutMix and Default. This reinforces its potential for robust and reliable generalization in external datasets.

The results show a clear correlation between increasing the blending parameter α in CIM and improved performance. CIM α = 0.8 consistently achieves the highest metrics across both datasets, with reduced variability, indicating stronger consistency in predictions. This suggests that higher α values enhance feature representation by providing richer augmented data while maintaining structural integrity.

Compared to the default CIM (no blending), CIM with blending demonstrates superior performance, particularly in sensitivity and F1 score, highlighting the value of blending in capturing complex lesion features. Blending at α = 0.8 proves especially effective, driving the best results in these specialized medical imaging tasks.

Beyond the improvements observed over MixUp and CutMix, it is important to contextualise CIM within the broader landscape of recent augmentation strategies. In our experiments, the naive “random lesion pasting” baseline provides a direct proxy for simple copy–paste methods widely used in natural-image literature and increasingly adopted in biomedical imaging. The consistent superiority of CIM over this baseline highlights the need for anatomically informed region selection and seamless Poisson-based blending—two aspects particularly relevant in GI imaging, where mucosal texture, colour gradients, and subtle lesion morphology strongly influence diagnostic interpretation. While more advanced generative approaches (e.g., GAN-based synthesis, diffusion models, or transformer-driven augmentation) offer alternative avenues for data expansion, these techniques typically demand large datasets, introduce limited control over lesion placement and morphology, and may produce hallucinated artefacts that compromise clinical trust. These constraints make them less suitable for highly localised tasks such as IM and dysplasia detection. By contrast, CIM provides a lightweight, interpretable, and anatomically coherent augmentation strategy, enabling fine-grained lesion preservation without the overhead of generative modelling.

Following the performance analysis, we examined where the models focus when making predictions by analyzing Grad-CAM heatmaps (Fig. 10). Our approach showed a stronger alignment with medical annotations, indicating that it is more aware of lesion features in both IM and dysplasia. This is likely due to CIM’s ability to generate more realistic augmented images that preserve key features, unlike CutMix, which blends images and may dilute critical regions.

To quantify this, we assessed the overlap between the heatmap-generated regions and the lesion ground truth annotation using Dice and IoU scores (Table 2). CIM consistently outperformed both Default and CutMix in these metrics. For IM, CIM achieved a Dice score of 0.414 ± 0.228 and IoU of 0.288 ± 0.191, while for dysplasia, CIM achieved Dice of 0.354 ± 0.205 and IoU of 0.235 ± 0.161. These results highlight that CIM’s augmented images better maintain the structure of the lesions, leading to a more precise and clear focus on relevant features, more correlated to the clinical annotations.

**Table 2:** Dice and IoU Scores for the overlay of Grad-Cam heatmaps with IM and dysplasia medical annotation.

	IM		Dysplasia	
	Dice	IoU	Dice	IoU
Default	0.399 ± 0.230	0.276 ± 0.187	0.316 ± 0.186	0.203 ± 0.142
CutMix	0.406 ± 0.224	0.280 ± 0.183	0.313 ± 198	0.203 ± 0.153
CIM	0.414 ± 0.228	0.288 ± 0.191	0.354 ± 205	0.235 ± 0.161

### Generalization capabilities

The models were trained on one dataset and evaluated on external test data, which involved different conditions, equipment, and potential environmental factors. Despite these variations, CIM with α = 0.8 demonstrated superior generalization capabilities compared to both Default and CutMix (Fig. 9). It consistently outperformed CutMix in most metrics, including Accuracy, Specificity, F1 Score, and AUC, while showing similar performance in Sensitivity. CIM’s higher mean values and lower variability across folds underscore its robustness and stability, making it a more reliable method for generalizing to unseen data compared to CutMix. This reinforces CIM’s potential for consistent performance across diverse scenarios.

The analysis of generalization capabilities using Repeated Measures ANOVA revealed no significant differences in Accuracy, Sensitivity, F1 Score, and AUC across the methods. However, significant differences were observed in Specificity (*p* = 0.0392), with CIM α = 0.8 and Default outperforming CutMix, indicating that both methods are slightly more effective at minimizing false positives. Although Precision showed no significant differences (*p* = 0.0711), CutMix exhibited higher variability, suggesting challenges in maintaining consistent precision across different folds.

Post-hoc pairwise comparisons for Specificity revealed that CIM α = 0.8 performed similarly to Default (*p* = 0.0203, BF10 = 4.231, Hedges’ g = 2.09), with strong evidence supporting its reliability. Comparisons between CIM α = 0.8 and CutMix (*p* = 0.1035, BF10 = 1.33, Hedges’ g = 1.36) showed moderate evidence favouring CIM as more stable in reducing false positives. Finally, CutMix and Default showed no significant difference (*p* = 0.7468), suggesting that CutMix lags behind CIM but is comparable to Default in Specificity. This reinforces CIM’s overall effectiveness and stability in generalization when applied to external datasets.

**Fig. 11:**
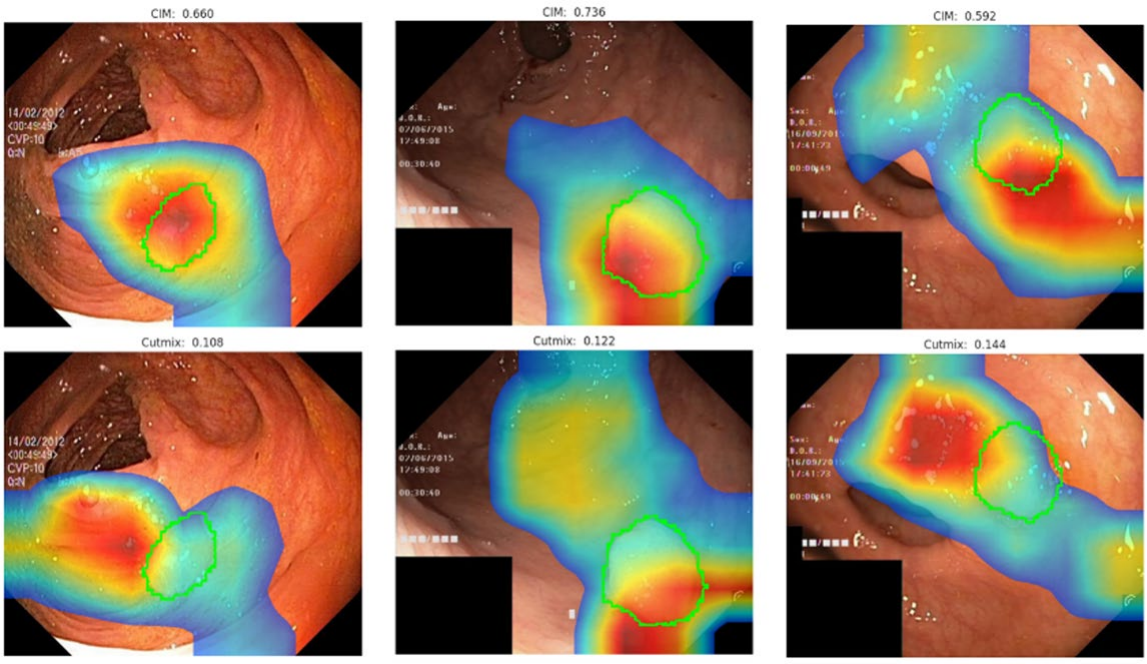
Grad-Cam heatmap examples for polyp endoscopy image classification.

For polyp detection, Grad-CAM analysis revealed that while both CIM and CutMix focused near lesion annotations, CutMix showed slightly better heatmap overlay with the annotated regions (Fig. 11). However, CIM outperformed CutMix in the classification task, as demonstrated in the results.

Quantitatively, CutMix achieved slightly higher segmentation metrics, with a Dice of 0.265 ± 0.194 and IoU of 0.169 ± 0.146, compared to CIM’s Dice of 0.257 ± 0.189 and IoU of 0.163 ± 0.140 (Table 3). The Default method performed worst in both segmentation and classification.

These findings highlight CIM’s superior classification capability, despite CutMix’s edge in overlaying the heatmaps over the lesion ground truth annotation, reinforcing CIM’s utility for tasks prioritizing accurate polyp classification.

**Table 3:** Dice and IoU Scores for the overlay of Grad-Cam heatmaps with polyp medical annotation.

Default	Dice	IoU
	0.242 ± 0.184	0.152 ± 0.134
CutMix	0.265 ± 0.194	0.169 ± 0.146
CIM	0.257 ± 0.189	0.163 ± 0.140

While CIM demonstrates consistent improvements over state-of-the-art augmentation strategies, several limitations should be acknowledged. First, CIM relies on unsupervised region-selection (MeanShift + SLIC) to identify biologically plausible placement areas. Although effective across the datasets studied, this process may generate suboptimal ROIs when mucosal texture is highly irregular or when illumination varies strongly across the field of view. Second, although Poisson-based blending substantially increases realism, lesion integration may still be imperfect under extreme lighting or colour shifts, potentially introducing subtle artefacts. Third, our evaluation was conducted on a single backbone architecture (ResNet50), selected deliberately to isolate the effect of augmentation rather than architectural variability. While this choice supports methodological clarity, future work should assess CIM on diverse architectures to further validate its generality. Finally, the present study focuses on binary classification tasks; extending CIM to multi-class or multi-lesion scenarios would provide a more comprehensive assessment of its applicability in real clinical settings.

### Conclusions and future work

This work introduced Cut Instance Mixing - CIM as a novel, domain-specific data augmentation method created for the challenges of GI endoscopy. By addressing the limitations of existing techniques, CIM improves model robustness and sensitivity, particularly in detecting subtle and irregular lesions like IM and dysplasia. Through biologically relevant augmentation strategies and advanced blending techniques, CIM delivers realistic training samples that enhance model generalization and classification accuracy.

Our experimental results demonstrate that CIM outperforms standard methods like MixUp, CutMix and simple random lesion copy-paste across multiple datasets and evaluation scenarios. CIM’s ability to maintain robust performance on external datasets further underscores its clinical applicability, where data is collected from different environments and conditions, compared to the data used to train the DL model.

Future work will focus on transforming CIM into a fully automated and intelligent system. This involves consolidating its functionalities into a unified framework capable of performing two critical tasks: (1) automatically identifying biologically relevant regions of interest directly from endoscopic images or another type of image, thereby eliminating the need for manual or semi-automatic segmentation, and (2) dynamically optimizing the blending degree for lesion integration based on the specific context of the target image, guided by patterns learned from training data. These advancements aim to streamline the augmentation process, minimize computational overhead, and further enhance the realism and effectiveness of augmented samples, ensuring improved performance across diverse medical imaging scenarios.

Additionally, future research will extend the assessment of CIM beyond binary classification to multi-class and multi-lesion scenarios, enabling evaluation in more clinically representative settings. We also aim to test CIM across multiple architectures, including lightweight CNNs, transformer-based models, and emerging hybrid vision backbones, to validate its architecture-agnostic properties. Beyond classification, applying CIM to segmentation and detection tasks will help determine its broader utility for spatially complex problems.

## Data Availability

The datasets generated and/or analysed during the current study are not publicly available due to patient confidentiality restrictions, but are available from the corresponding author on reasonable request. All patients were informed about the research purposes and signed clarified written consents, and the Ethical Committee of IPO-Porto approved the data usage for this study (Parecer CES. 154/023). All methods were performed in accordance with the relevant guidelines and regulations.
